# Flavonoid Versus Artemisinin Anti-malarial Activity in *Artemisia annua* Whole-Leaf Extracts

**DOI:** 10.3389/fpls.2019.00984

**Published:** 2019-07-30

**Authors:** Tomasz Czechowski, Mauro A. Rinaldi, Mufuliat Toyin Famodimu, Maria Van Veelen, Tony R. Larson, Thilo Winzer, Deborah A. Rathbone, David Harvey, Paul Horrocks, Ian A. Graham

**Affiliations:** ^1^Centre for Novel Agricultural Products, Department of Biology, University of York, York, United Kingdom; ^2^Institute for Science and Technology in Medicine, Keele University, Keele, United Kingdom; ^3^School of Medicine, Keele University, Keele, United Kingdom; ^4^Biorenewables Development Centre, Dunnington, United Kingdom

**Keywords:** malaria, *Artemisia annua*, artemisinin, flavonoids, *Plasmodium falciparum*, chalcone isomerase

## Abstract

Artemisinin, a sesquiterpene lactone produced by *Artemisia annua* glandular secretory trichomes, is the active ingredient in the most effective treatment for uncomplicated malaria caused by *Plasmodium falciparum* parasites. Other metabolites in *A. annua* or related species, particularly flavonoids, have been proposed to either act as antimalarials on their own or act synergistically with artemisinin to enhance antimalarial activity. We identified a mutation that disrupts the CHALCONE ISOMERASE 1 (CHI1) enzyme that is responsible for the second committed step of flavonoid biosynthesis. Detailed metabolite profiling revealed that *chi1-1* lacks all major flavonoids but produces wild-type artemisinin levels, making this mutant a useful tool to test the antiplasmodial effects of flavonoids. We used whole-leaf extracts from *chi1-1* and mutant lines impaired in artemisinin production in bioactivity *in vitro* assays against intraerythrocytic *P. falciparum* Dd2. We found that *chi1-1* extracts did not differ from wild-type extracts in antiplasmodial efficacy nor initial rate of cytocidal action. Furthermore, extracts from the *A. annua cyp71av1-1* mutant and RNAi lines impaired in amorpha-4,11-diene synthase gene expression, which are both severely compromised in artemisinin biosynthesis but unaffected in flavonoid metabolism, showed very low or no antiplasmodial activity. These results demonstrate that *in vitro* bioactivity against *P. falciparum* of flavonoids is negligible when compared to that of artemisinin.

## Introduction

Malaria is one of the most prevalent infectious diseases with 219 million cases and 435,000 deaths reported in 2017 (World Health Organization [WHO], 2018). The WHO recommends the use of artemisinin-based combination therapies (ACTs) for treatment of uncomplicated malaria caused by the *Plasmodium falciparum* parasite (World Health Organization [WHO], 2018). ACTs consist of fast-acting and stable artemisinin derivatives, such as artesunate, co-formulated with a different class of drug to reduce the emergence of resistance and increase treatment efficacy (Petersen et al., 2011). The main source of the sesquiterpene artemisinin is currently the medicinal plant *Artemisia annua*, which has achieved a yield of 1.5% dry leaf weight through breeding (Townsend et al., 2013). Additionally, a semi-synthetic alternative has been developed through precursor biosynthesis in yeast and chemical conversion to artemisinin (Peplow, 2016).

*Artemisia annua* accumulates artemisinin together with a wide range of secondary metabolites in the extracellular subapical cavity of glandular secretory trichomes, specialized 10-cell structures on the surfaces of aerial tissues (Brown, 2010; Lange, 2015; Czechowski et al., 2018). This wide range of metabolites has led to speculation that perhaps other compounds in *A. annua* or related species might act as antimalarials or potentially enhance the antimalarial activity of artemisinin. Therefore, several groups have tried to isolate and identify metabolites from *A. annua* and related species that might function as antimalarials (O’Neill et al., 1985; Elford et al., 1987; Liu et al., 1989, 1992; Cubukcu et al., 1990; Mueller et al., 2000; Bhakuni et al., 2001; Kraft et al., 2003).

Recent publications have reported that *A. annua* whole-plant preparations are more effective than artemisinin alone (not ACTs) in treating rodent malaria (Elfawal et al., 2012) and reducing the development of resistance (Elfawal et al., 2015), and that whole-plant preparations may be effective in treating artesunate-resistant malaria patients (Daddy et al., 2017). These results suggest that *A. annua* produces metabolites that might act together with artemisinin and thus whole-plant preparations have been proposed as replacement treatments for ACTs (Weathers et al., 2014). In particular, flavonoids have been singled out as the likely synergistic metabolites (Elfawal et al., 2012, 2015; Weathers et al., 2014; Daddy et al., 2017) mainly because there is some evidence that they may improve the antimalarial activity of artemisinin *in vitro* (Elford et al., 1987; Liu et al., 1989, 1992; Ferreira et al., 2010).

Flavonoids are a diverse class of plant and fungal secondary metabolites with over 6500 different flavonoid products described from the secondary metabolism of various plant species (Ververidis et al., 2007). Flavonoid biosynthesis starts from primary metabolism precursors: phenylalanine and malonyl-CoA. Phenylalanine is used to produce 4-coumaroyl-CoA which is then combined with malonyl-CoA by chalcone synthase to yield the two-phenyl ring backbone common to all chalcones. A key step in flavonoid synthesis is the conjugate ring-closure of chalcones catalyzed by chalcone-flavanone isomerase (CHI), which results in the three-ringed structure of a flavone. The phenylpropanoid metabolic pathway contributes a series of enzymatic modifications that yield flavanones, dihydroflavonols, and eventually anthocyanins. Many products can be derived from this pathway including flavonols, flavan-3-ols, proanthocyanidins (tannins) and a host of other various polyphenolics (Ververidis et al., 2007). Flavonoids have been classified according to the position of the linkage of the aromatic ring to the benzopyrano moiety into four classes: major flavonoids (2-phenyl benzopyrans), which include flavonols, flavonones, flavanonols, flavones, anthocyanins and anthocyanidines; isoflavonoids (3-benzopyrans), which contain isoflavanons, isoflavanones and isoflavanonols; neo-flavonoids (4-benzopyrans) which include neoflavenes and 4-arylcoumarins; and finally, minor flavonoids which include aurones, auronols, 2′OH chalcones and 2′OH dihydrochalcones (Ververidis et al., 2007).

In the present work we report an *A. annua* loss-of-function mutation of the trichome-specific *CHALCONE ISOMERASE 1* (*CHI1*) gene. Levels of all major flavonoids in the *chi1-1* mutant were reduced to undetectable levels. We used *chi1-1* to test the antimalarial effects of flavonoids. We extended the bioassays to include whole-leaf extracts from *A. annua* silenced in amorpha-4,11-diene synthase (*AMS*), which encodes the enzyme that catalyzes the first committed step of artemisinin biosynthesis (Catania et al., 2018), and the *cyp71av1-1* mutant, impaired in the second committed step of artemisinin biosynthesis (Czechowski et al., 2016). The *AMS* silenced line has dramatically reduced artemisinin production (5% of the wild-type levels) and accumulates the sesquiterpene precursor farnesyl pyrophosphate in trichomes (Catania et al., 2018). *cyp71av1-1* completely abolishes artemisinin production and redirects the artemisinin pathway to the synthesis of arteannuin X, a novel sesquiterpene epoxide (Czechowski et al., 2016). Both the *AMS* silenced and *cyp71av1-1* mutant lines produce wild-type levels of major flavonoids. We have performed a comparative analysis of whole-leaf extracts from *chi1-1*, the *AMS* silenced line, *cyp71av1-1*, and wild-type *A. annua* in *in vitro P. falciparum* Dd2 kill assays (Ullah et al., 2017) to determine the antiplasmodial efficacy and initial cytocidal activity of these extracts.

## Materials and Methods

### Plant Material

For wild-type plant material we used the Artemis F1 hybrid variety of *A. annua* developed by Mediplant (Conthey, Switzerland), obtained by crossing C4 and C1 parental lines of East Asia origin (Delabays et al., 2001). Seeds were sown in 4-inch pots filled with Levington modular compost and grown in a glasshouse under long-day conditions (16-h day/8-h night) at 17–22^∘^C for 12 weeks.

### RNA Isolation and Semi-Quantitative RT-PCR

Total RNA was isolated from eight *A. annua* tissues: meristems, cotyledons, trichomes, young leaves, expanded leaves, mature leaves, stems, and flowers as previously described (Graham et al., 2010) and quantified using the NanoDrop 8000 (NanoDrop products, Wilmington, DE, United States). 5 μg of total RNA was digested with RQ1 RNase-free DNase (Promega, United Kingdom) according to the manufacturer’s protocol. 1st strand cDNA synthesis was performed using 2.5 μg of DNaseI digested RNA with oligo dT(18) primers and SuperScript^TM^ II Reverse Transcriptase (Thermo Fisher, United Kingdom) according to manufacturer’s protocols. 3 μL of the first strand cDNA was used for PCR amplification using the following gene specific primers: CHI1_For: 5′-TGGCAACACCACCTTCAGC TACC-3′ (left), CHI1_Rev: 5′-GTTGTGAAGAGAATAGAG GCG-3′ (right), CHI2_For: 5′-ATGGCTAAGCTTCATTCCTCC AC-3′ (left), CHI2_Rev: 5′-CAGGTATGATACCATCTCTA GC-3′ (right), CHI3_For 5′-CTGGAGCAATTCCCAGATC AG-3′ (left), CHI3_Rev 5′-AGAATGTTTTGCCATCAACATC TC-3′ (right), Ubiquitn_For: 5′-GTCGGCTAATGGAGAAG ACAAGAAG-3′ (left) and Ubiquitn_Rev: 5′-GAAAGCA CGACCAGATTCATAGC-3′ (right) using GoTaq^TM^ *Taq* polymerase (Promega, United Kingdom). The PCR program used to amplify the target sequences was: 94^∘^C, 2 min; followed by 10 cycles of “touch down”: 94^∘^C, 30 sec; 65^∘^C (−1^∘^C/cycle); 72^∘^C, 1 min, followed by 20 cycles of 94^∘^C, 30 sec; 55^∘^C, 30 sec; 72^∘^C, 1 min, followed by a final extension at 72^∘^C for 5 min. 10 μL of PCR product was resolved on 1% agarose gels. Predicted product sizes for each gene are: 466 bp for *CHI1*, 520 bp for *CHI2*, 507 bp for *CHI3*, and 454 bp for *UBQ*.

### *chi1-1* Mutant Isolation and Characterization

An ethyl methanesulfonate-mutagenized *A. annua* population was established as described before (Graham et al., 2010; Czechowski et al., 2016). Screening of the self-fertilized M2 population was performed as previously described (Czechowski et al., 2016) with the following modifications. DNA was isolated from 30 to 50 mg of fresh leaf material harvested from individual 4 to 6-week-old M2 plants, using the BioSprint 96 system (Qiagen, Hilden, Germany) according to the manufacturer’s protocol. DNA was quantified fluorometrically using Hoechst 33258 dye and a plate reader (Fuoromax, United Kingdom). DNA samples were normalized to 5 ng/μL using the Freedom EVO^®^ 200 workstation (Tecan United Kingdom Ltd.) and arranged in four-fold pools for reverse genetic screening. The full-length genomic DNA sequence of the Artemis *A. annua CHI1* gene for TILLING was obtained by PCR using gene-specific primers designed based on Gene Bank-deposited sequence EZ246664. A 937-bp fragment of CHI1 was amplified in a two-step PCR reaction. The first step was carried out with unlabeled primers: 5′-TGGCAACACCACCTTCAGCTACC-3′ (left) and 5′-CTGTGGTTGCTTTCTCATCAAAATGG-3′ (right) on 12.5 ng of pooled gDNA in 10 μL volumes. Nested PCR and labeling with IR dyes were performed on a 1/10 dilution of the first PCR with a mixture of unlabeled M13-tailed primers (5′-TGTAAAACGACGGCCAGTCGACAGCAACTAGTAATGG TAAACTG-3′ (left) and 5′-AGGAAACAGCTATGACCACAT AAGATCTGAAAGTCTTGAAGCC-3′ (right), and with M13 left primer (5′-TGTAAAACGACGGCCAGT-3′) labeled with IRDye700 and M13 right primer (5′-AGGAAACAGCTATGACC AT-3′) labeled with IRDye 800 (MWG, Ebersberg, Germany). Heteroduplex formation, CEL I nuclease digestion and analysis on the LI-COR 4300 DNA sequencer platform were carried out as previously described (Till et al., 2006). All mutants found on the TILLING gels were verified by Sanger sequencing of both DNA strands of PCR-amplified fragments using the following primers: 5′-GCAATAATGCTATGTGTTGGTGC-3′ (left) and 5′-CACAATGTTTGCAGCTTCAGGTATG-3′ (right). Two segregating M3 mutant populations were obtained by crossing M2 siblings that were heterozygous for the *chi1-1* mutation.

### KASP^TM^ SNP Assay for *chi1-1* Mutation Status

Twenty nanograms of DNA was used for 10 μL KASPar assay reactions containing: 1 × KASP V4.0 low ROX master mix (LGC Genomics); a concentration of 167 nM of each of the two allele-specific primers: chi1-1_ForC: 5′-GAAG GTGACCAAGTTCATGCTCAATGATACTACCATTAACTGGT AAGC-3′ and chi1-1_ForT: 5′-GAAGGTCGGAGTCAACGGAT TGACAATGATACTACCATTAACTGGTAAGT-3′ and 414 nM universal primer chi1-1_Rev 5′-CTCCAACGCACATTTCAGA CACCTT-3′, according to the manufacturer’s recommendations. Allelic discrimination runs and allelic discrimination analysis were performed on Viia7 system (Life Technologies Ltd.) according to the manufacturer’s recommendations.

### Metabolite Analysis by UPLC-and GC-MS

Plants were grown from five cuttings from each genotype and metabolic profiles were generated from 10 to 50 mg FW pooled samples of leaves at different developmental stages: 4–6 (counting from the apical meristem) representing the young stage; leaves 11–13 representing the mature, expanded stage and three leaves taken just above first senescing leaves representing old leaves. Fresh leaf samples were stored at −80^∘^C. Trichome-specific metabolites were extracted as described previously (Czechowski et al., 2016) and analyzed by UPLC-MS as previously described (Graham et al., 2010; Czechowski et al., 2016). Dry leaf material was obtained from 14-week-old plants, cut just above the zone of senescing leaves and dried for 14 days at 40^∘^C. Leaves were stripped from the plants, and leaf material sieved through 5 mm mesh to remove small stems. Metabolite extractions from 10 mg of the dry leaf material and UPLC-MS analysis were performed as previously described (Graham et al., 2010; Czechowski et al., 2016). Number of the biological replicates measured was as follows: young and mature wild-type leaves *n* = 49, old wild-type leaves *n* = 60, dry wild-type leaves *n* = 21; young-, mature- and old heterozygous *chi1-1* leaves *n* = 94, dry heterozygous *chi1-1* leaves *n* = 37; young, mature and old homozygous *chi1-1* leaves *n* = 63, dry heterozygous *chi1-1* leaves *n* = 32. The experiments comparing trichome vs. whole-leaf metabolites were performed on leaves 14–16 harvested from five individuals grown from cuttings (*n* = 5). Trichome-specific metabolites were first extracted from the fresh mature leaves as described above. The remaining leaf material was washed three times with 500 μL of chloroform and solvent removed by pipetting. Leaf tissue was ground to a fine powder in TissueLyser II (Qiagen, United Kingdom), extracted and quantified by UPLC-MS. GC-MS analysis was performed on the same dipped and ground-leaf extracts as described before (Czechowski et al., 2016). To evaluate method suitability for detecting flavonoids, comparative extracts of dry material were made with either 9:1 chloroform:ethanol (v/v; used throughout this study) vs. 85:15 methanol:water (v/v; typically used to extract polar flavonoids from plant material). These extracts were then separated on an extended UPLC gradient (starting conditions modified to 100% of aqueous solvent A), to avoid any potentially highly polar flavonoids being lost in the void volume.

### Whole-Leaf Extraction for *P. falciparum* Kill Rate Assays

Fourteen-week-old plants were cut above the area of senescing leaves and dried for 14 days at 40^∘^C. Leaves were separated from the rest of the dry plants and sieved through 5-mm mesh to remove small stems. Dry leaves were stored long term in a humidity-controlled cabinet at 4^∘^C. For whole-leaf extracts, 1 g dry leaves was ground to a fine powder and extracted in 9:1 chloroform and ethanol solution overnight, centrifuged at 4,700 rpm for 20 min and the supernatant was filtered through Wattman paper. An aliquot was taken for quantification by UPLC-MS. The solvent was evaporated until only an oily residue remained and re-suspended in DMSO to reach a final concentration of 5 mg/ml artemisinin (or to reach a casticin concentration equivalent to that of the wild type in the *AMS* RNAi line, or equivalent to heterozygous *cyp71av1-1* in homozygous *cyp71av1-1*).

### *In vitro Plasmodium falciparum* Assays

The *in vitro* screening of antiplasmodial activity of extracts was carried out starting with trophozoite stage (24–32 h post infection) intraerythrocytic stages of the *P. falciparum* Dd2 strain using a 48 h (one complete cycle of intraerythrocytic development) Malaria Sybr Green I Fluorescence assay as previously described (Smilkstein et al., 2004; Ullah et al., 2017). The mean percentage growth ± StDev (*n* = 9 from three independent biological repeats) was plotted against log_10_-transformed drug concentration and a non-linear regression (sigmoidal concentration–response/variable slope equation) in GraphPad Prism v5.0 (GraphPad Software, Inc., San Diego, CA, United States) used to estimate the 50% effective concentration (EC_50_) and the 95% confidence intervals.

Determination of the relative initial cytocidal activity against trophozoite stage intraerythrocytic stages of the *P. falciparum* Dd2^luc^ (Wong et al., 2011) were carried out using the Bioluminescence Relative Rate of Kill assay as described (Ullah et al., 2017). All assays were carried out over 6 h using a 9 × EC_50_ to 0.33 × EC_50_ concentration series. The mean ± StDev bioluminescence signal, normalized to an untreated control, are plotted (*n* = 9 from three biological repeats) and compared to the benchmark standards of dihydroartemisinin (DHA), chloroquine (CQ), mefloquine (MQ) and atovaquone (ATQ). Stock solutions of atovaquone (10 mM in DMSO), chloroquine (100 mM in deionized water), dihydroartemisinin (50 mM in methanol), and mefloquine (50 mM in DMSO) were made (Sigma-Aldrich) and stored at −20^∘^C. In all experiments, the maximum final concentration of solvent was 0.6% (v/v).

## Results

### Isolation and Characterisation of a *CHI1* Mutant Impaired in Flavonoid Biosynthesis

**FIGURE 1:**
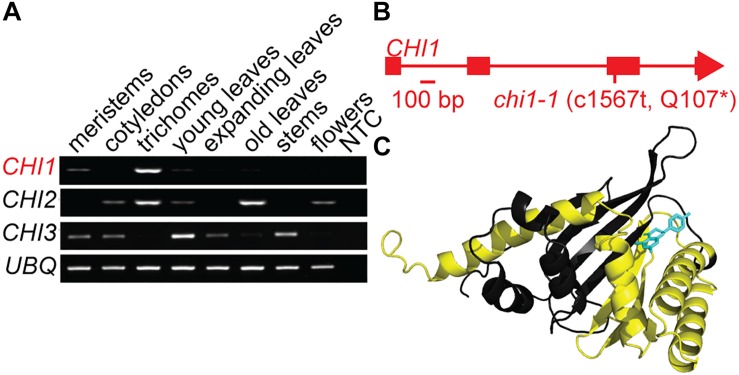
Discovery and characterization of the *chi1-1* mutation. **(A)** *Artemisia annua CHI1*, *CHI2* and *CHI3* expression in meristems, cotyledons, young leaf trichomes, young leaves, fully expanded leaves, mature leaves, stems, flowers, and no template control (NTC) were determined by semi-quantitative PCR. *UBQ* (Putative ubiquitin-like protein, GQ901904) was used as a loading control. **(B)** Gene schematic of *CHI1* indicates the position of the *chi1-1* mutation. **(C)** The *A. annua* CHI1 protein structure was modeled by I-TASSER (Yang et al., 2015) on the 10 most closely related structural analogs. The parts of the structure expected to be missing in the *chi1-1* mutant are highlighted in yellow, naringenin (enzyme product) bound to CHI1 is shown in blue.

Casticin and other polymethoxylated flavonoids accumulate in leaf and flower trichomes of the *A. annua* Artemis variety, some to high levels comparable to those of artemisinin (Czechowski et al., 2016, 2018). We previously identified three putative *CHI* genes using *A. annua* transcriptome data (Graham et al., 2010). *CHI1* is expressed in young leaf and flower bud trichomes whereas *CHI2* is expressed in young and mature leaf trichomes and *CHI3* is expressed most highly in meristems and cotyledons (Graham et al., 2010). Further quantitative RT-PCR-based expression profiling, extended to other tissues, revealed that *CHI1* expression is the most trichome specific of the three genes tested, whereas *CHI2* is more generally expressed in several tissues and *CHI3* expression is not detected in trichomes (Figure 1A). The 229 amino acid-long predicted protein sequence for CHI1 is most similar to CHI characterized in other organisms than it is to the other two putative CHI proteins we previously identified from *A. annua* (Supplementary Figure S1A; Jez et al., 2000). Amino acid sequence alignment of CHI homologs shows that CHI2 and CHI3 are missing a number of highly conserved residues including those required for substrate binding (Supplementary Figure S1A; Jez et al., 2000). In contrast CHI1 contains all of the conserved residues, suggesting that it is the only one of the three CHI homologs from *A. annua* that produces a functional chalcone isomerase enzyme.

Using an established ethyl methanesulfonate-mutagenized population of *A. annua* (Czechowski et al., 2016) we performed a TILLING screen of the single-copy *CHI1* gene that resulted in an allelic series of five mutants, including three with intronic mutations, one with a silent mutation and one with a nonsense mutation that created a C1567 to T transition in the third exon of *CHI1* (Figure 1B and Supplementary Figure S1B). The latter mutation, which we designate *chi1-1*, gave a predicted change of amino acid Gln107 in the polypeptide to a stop codon that would result in a major truncation of the enzyme and loss of most of the putative substrate-binding site (Figure 1C and Supplementary Figure S1A). CHI is a functional monomer and residues that are important for substrate binding and the active site in other species lie beyond the residue corresponding to *A. annua* CHI1 Q107 (Figure 1C and Supplementary Figure S1A; Jez et al., 2000), which suggested the truncation would result in a complete loss of CHI function.

In order to investigate the effects of the *chi1-1* mutation on artemisinin and flavonoid biosynthesis we analyzed three leaf developmental stages: young (leaves 4–6 as counted down from the apical meristem), mature (leaves 11–13) and old (3 leaves preceding the first senescing leaves). To generate material for this analysis we performed two crosses of heterozygous *chi1-1* M2 siblings originating from a self-fertilized M1 individual and performed DNA marker-based selection of wild type (WT) and heterozygous and homozygous *chi1-1* individuals from the segregating M3 population using the KASP^TM^ SNP assay. We observed a strong segregation distortion from the expected 1:2:1 (WT:heterozygous:homozygous mutation) in both M3 populations. The first cross resulted in 30 individuals of which 24 were heterozygous and 6 homozygous for the *chi1-1* mutation whereas the second cross resulted in 54 individuals of which 36 were heterozygous and 18 homozygous for the *chi1-1* mutation, but we could not identify segregating wild-type individuals. Such segregation distortion is not unusual for *A. annua*, which naturally outcrosses, and has been reported for the populations coming from self-fertilized individuals (Graham et al., 2010). In the absence of any segregating M3 wild–type individuals we used non-mutagenized *Artemis* F1 as wild type for metabolic profiling.

**FIGURE 2:**
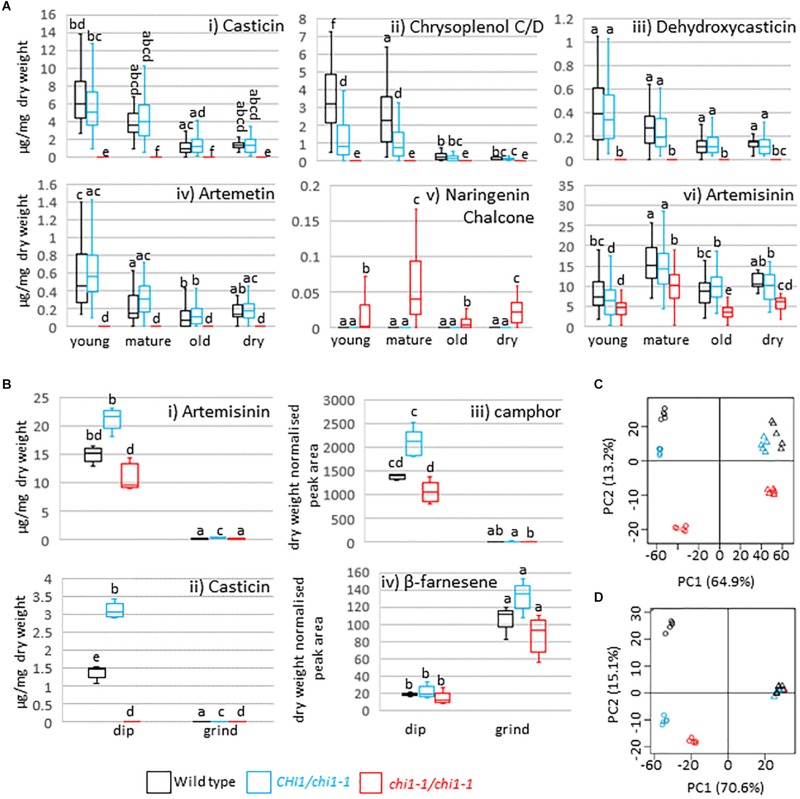
Effects of the *chi1-1* mutation on the metabolite profile of *Artemisia annua*. Box and whisker plots showing levels of **(A)** four major flavonoids, putative naringenin chalcone and artemisinin as measured by UPLC-MS in young (leaves 1–5 as counted from the apical meristem), mature (leaves 11–13), old (three leaves above first senescing leaf) and dry (oven-dried) leaf material harvested from 12 to 14-week-old plants of the *Artemis* wild type (black), heterozygous (blue) and homozygous *chi1-1* mutant (red) and **(B)** selected flavonoids, sesquiterpenes and monoterpenes in the extracts from dipped (dip) or ground leaf material for the wild type (black) and heterozygous (blue) and homozygous (red) *chi1-1* mutant. Metabolite concentrations measured by GC- or UPLC-MS **(A,B)** are expressed as a proportion of the residual dry leaf material following extraction. Groups not sharing letters representing Tukey’s range test results indicate statistically significant differences (*p* < 0.05). Each box is represented by minimum of 20 **(A)** or by five **(B)** biological replicates. **(C,D)** Principal component analysis of 83 UPLC-MS identified peaks **(C)** and of 58 GC-MS identified peaks **(D)** from dipped and ground leaf material from wild type (black) and heterozygous (blue) and homozygous *chi1-1* (red). Dip leaf extracts are represented by circles and ground leaf extracts by triangles. Principal component analysis was performed on log-scaled and mean-centered data.

*CHI* disruption or suppression has previously been reported to result in discoloration and/or decreased flavonol levels in *Arabidopsis thaliana*, petunia, carnation, onion and tobacco (van Tunen et al., 1991; Shirley et al., 1992; Itoh et al., 2002; Kim et al., 2004; Nishihara et al., 2005) whereas petunia *CHI* overexpression leads to increased flavonol accumulation in tomato (Muir et al., 2001). *A. annua* produces the polymethoxylated flavonoids casticin, artemetin, chrysoplenetin, chrysosplenol-D, and cirsilineol (Bhakuni et al., 2001). Whereas the wild type and the *chi1-1* heterozygote produced similar amounts of casticin, chrysoplenol C, dehydroxycasticin and artemetin none of these flavonoids were detectable in homozygous *chi1-1* individuals (Figures 2Ai–iv). These results demonstrate that *chi1-1* is a null allele. Flavonoids in the wild type and heterozygous *chi1-1* are most abundant in young, followed by mature and old leaves (Figure 2A). Noteworthy, *chi1-1* accumulated a compound with an m/z ratio of 273.0757 that was not detectable in the wild type or the *chi1-1* heterozygote (Figure 2Av). The UPLC-MS profile of this compound suggests it represents the molecular ion of naringenin chalcone (MW = 272.26 g/mol), the substrate of chalcone isomerase, which would be expected to accumulate in the *chi1-1* null mutant.

We initially devised our chloroform:ethanol extraction method to be optimal for artemisinin extraction, which has a logP of 2.8. Chloroform has a logP value of ∼2.3, which is also quite closely matched to the calculated logP values of *A. annua* methoxylated flavonoids (2.1–3.4, using structures reported by Ferreira et al., 2010). We compared our 9:1 chloroform:ethanol extraction method used throughout this study with a solvent more typically used for flavonoid extraction (85:15 methanol:water) by extracting WT and homozygous *chi1-1* dry material (Supplementary Table S4). The UPLC method was also extended so that the elution conditions at the start of the run were much more aqueous, to ensure that any polar flavonoids (if present) were not eluted in the void volume. Peaks were picked and identified according to our standard high-resolution accurate mass protocols, and additionally matched against formula hits for 40 previously reported flavone and flavonol compounds from *A. annua* (Ferreira et al., 2010). The results show, from dry material, that 142 peaks could be resolved of which only six potential flavonoids could be identified; all six of these compounds were extracted in both solvent systems, and were in fact best extracted in our standard chloroform:ethanol solvent (Supplementary Table S4). As expected, highly polar phenolic compounds such as scopolin and scopoletin (PubChem xlogP values of −1.1 and 1.5, respectively) extracted better in the methanolic solvent and could be resolved using the adapted UPLC method. All 40 flavonoids reported by Ferreira et al. (2010) have predicted xlogP values in the range −1.3–3.5, so we would expect to detect these in the modified UPLC method, if present in any of the extracts. From this comparison we conclude that our chloroform:ethanol extraction solvent is sufficient to extract the full suite of flavonoids present in the various *A. annua* genotypes used in the present study, which all derive from the F1 Artemis commercial variety (Delabays et al., 2001) which serves as the wild type in the current study. In a detailed metabolite analysis of high- and low- artemisinin-producing chemotypes of *A. annua*, which involved both MS and NMR based detection and identification we found similarly low numbers of flavonoids (Czechowski et al., 2018). We note that the much larger number of flavonoids reported in the review by Ferreira et al. (2010) are based to an extent on HPLC-UV analysis of *A. annua* material obtained from Yunnan Herbarium, China (Lai et al., 2007). Future work involving comparative metabolite analysis of different cultivars grown under identical conditions should help establish the basis of the difference in the numbers of flavonoids being reported in these different studies.

Finally, artemisinin levels were consistently decreased in all homozygous *chi1-1* leaf material types compared to heterozygous *chi1-1* and the wild type (Figure 2Avi). DHAA levels were simultaneously reduced in young leaves of homozygous *chi1-1* when compared to heterozygous *chi1-1* and the wild type (Supplementary Table S1). We also observed a mild reduction in the level of dihydroartemisinic acid tertiary allylic hydroperoxide in all leaf types of homozygous *chi1-1* when compared to heterozygous *chi1-1* and the wild type. On the other hand, levels of DHAA-derived 11,13-dihydroamorphanes such as dihydro-epi-deoxy arteannuin B, deoxyartemisinin, arteannuin I/J, arteannuin M/O and 11-hydroxy-arteannuin remained unchanged in homozygous *chi1-1* (Supplementary Table S1).

To further confirm the specificity of the effects of the *chi1-1* mutation on trichomes, we analyzed metabolites in trichomes and leaves separately. Fresh mature leaves were dipped in chloroform to disrupt the trichomes and release the contents (dip), as previously described (Graham et al., 2010), and the remaining leaf material was ground, extracted and analyzed separately (ground leaves). Known trichome-specific compounds such as artemisinin, DHAA or camphor were found in extracts from the dip treatment but not in the post-dip ground leaf extracts (Figures 2Bi,iii and Supplementary Table S2), consistent with previous morphological studies (Duke et al., 1994) and the trichome-specific expression of the relevant biosynthetic pathway enzymes (Olsson et al., 2009; Graham et al., 2010; Olofsson et al., 2011; Soetaert et al., 2013). Casticin, chrysoplenol C/D, dehydroxycasticin and artemetin were also found in extracts from dip treatments but not in post-dip ground leaf extracts from heterozygous *chi1-1* or the wild type, but were completely absent in homozygous *chi1-1* dip and post-dip ground leaf extracts (Figure 2Bii and Supplementary Table S2). β-farnesene, germacrene-D, *trans-*caryophyllene and squalene were found mostly in post-dip ground leaf extracts (Figure 2Biv and Supplementary Tables S2, S3). This is consistent with the previous metabolite studies on gland bearing vs. glandless biotypes of *A. annua* (Tellez et al., 1999) and with the ubiquitous expression of the relevant terpene synthases in *A. annua* (Graham et al., 2010; Olofsson et al., 2011). A principal component analysis for 83 of the UPLC-MS (Figure 2C) and 58 of the GC-MS (Figure 2D) detectable metabolites revealed that homozygous *chi1-1* more strongly diverged from the wild type and heterozygous *chi1-1* in extracts from dip treatment, but less so in post-dip ground leaf extracts, where ground material clustered together. These findings suggested that the *chi1-1* mutant is mainly disrupted in trichome metabolism and that *CHI1* is needed for flavonoid synthesis specifically in trichomes.

### Flavonoids Do Not Contribute Antimalarial Activity in Whole-Leaf Extracts

**FIGURE 3:**
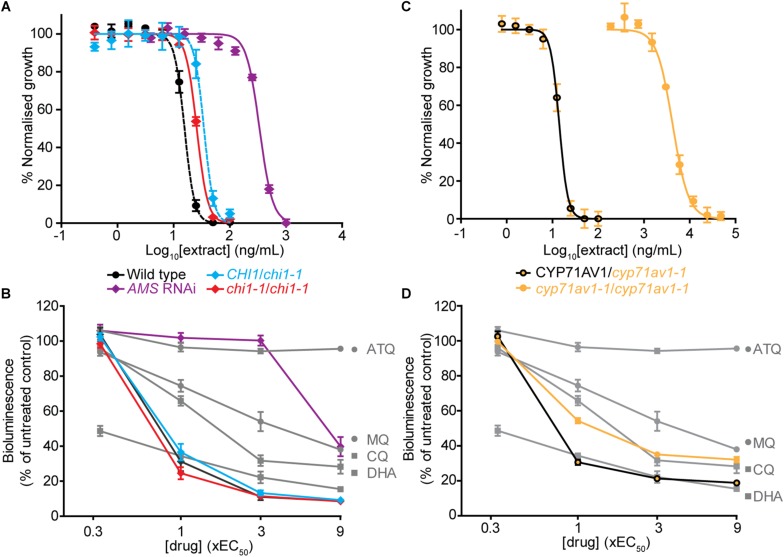
Comparison of *in vitro* antiplasmodial activity of leaf extracts from *Artemisia annua* wild type, mutant and antisense lines with altered flavonoid and artemisinin content. **(A,C)** Log concentration-normalized response curves of *Plasmodium falciparum* parasites after 48 h of treatment with extracts used to determine the EC_50_ (50% effective concentration of extract needed to inhibit growth of the *P. falciparum* parasites) of the indicated extracts. **(B,D)** Bioluminescent Relative Rate of Kill (BRRoK) assays to determine the initial (6 h) cytocidal action, compared to an untreated control after exposure to extracts of wild type, heterozygous and homozygous *chi1-1*, and the *AMS* silenced line **(B)** or heterozygous and homozygous *cyp71av1-1* **(D)** at multiples of the EC_50_ alongside dihydroartemisinin (DHA) > chloroquine (CQ) > mefloquine (MQ) > atovaquone (ATQ) benchmark controls. Error bars represent standard deviations from the means of three biological replicates.

The *chi1-1* line allowed for a direct comparison of *A. annua* extracts with and without flavonoids to evaluate the contribution of the cytocidal effects of these compounds on *Plasmodium* parasites *in vitro*. To evaluate whether there were changes from the potent and rapid cytocidal effects expected from artemisinin-containing extracts, the metabolites from wild type, and heterozygous and homozygous *chi1-1* extracts were quantified and re-suspended to the same artemisinin concentration (Table 1). The antiplasmodial activity against asexual intraerythrocytic stages of *P. falciparum* indicated that the effective concentration required to inhibit growth by 50% (EC_50_) was essentially the same, between 15 and 35 ng/ml for the wild type and heterozygous and homozygous *chi1-1* (Figure 3A and Table 1). We also performed an evaluation of the initial cytocidal activity of the same extracts using a Bioluminescence Relative Rate of Kill (BRRoK) assay (Ullah et al., 2017). Here, asexual intraerythrocytic stages of *P. falciparum* are exposed to multiples (0.33 to 9X) of EC_50_ of extract, or benchmark antimalarial drugs of a known order of rate of kill, for 6 h. This assay allows a compound/extract to be compared to fast cytocidal drugs like artemisinin, the derivative dihydroartemisinin and chloroquine; slower cytocidal drugs like mefloquine; and cytostatic drugs such as atovaquone (Ullah et al., 2017). When performing BRRoK assays, the three samples were indistinguishable from one another and most similar to dihydroartemisinin (the active metabolite of artemisinin compounds) in the concentration *v.* loss of bioluminescence plot (Figure 3B). These results indicate that flavonoids in the wild-type extracts did not alter the fast cytocidal activity of artemisinin in the samples.

**TABLE 1:** Artemisinin and flavonoid levels and antimalarial efficacy of plant extracts.

	**Artemisinin (mg/mL)**	**Casticin (mg/mL)**	**Dehydroxycasticin (mg/mL)**	**Cirsilineol (mg/mL)**	**Chrysoplenol C (mg/mL)**	**Artemetin (mg/mL)**	**Total detected flavonoid (mg/mL)**	**EC_50_ (ng/mL) [95% CI]**
Wild type	5.00±0.80^b^	0.51±0.07^c^	0.09±0.01^c^	0.11±0.04^b^	0.004±0.003^a^	0.00^a^	0.71±0.12^c^	15.6 [14.5–16.8]
*chi1-1* het	5.00±0.28^b^	0.36±0.02^b^	0.042±0.009^b^	0.00^*a*^	0.005±0.002^a^	0.00^a^	0.40±0.03^b^	34.6 [31.7–37.9]
*chi1-1* hom	5.00±0.44^b^	0.00^a^	0.00^a^	0.00^a^	0.00^a^	0.00^a^	0.00^a^	25.7 [25.1–26.4]
*AMS* silenced line	0.062±0.007^a^	0.50±0.06^c^	0.021±0.007^*ab*^	0.006±0.002^a^	0.00^a^	0.12±0.01^c^	0.65±0.08^c^	350.4 [303.1–405.1]
*cyp71av1-1* het	5.00±0.55^b^	0.69±0.04^d^	0.15±0.02^d^	0.00^a^	0.074±0.012^b^	0.00^a^	0.91±0.05^d^	14.1 [13.5–14.7]
*cyp71av1-1* hom	0.00^a^	0.61±0.14^*cd*^	0.31±0.05^e^	0.00^a^	0.00^a^	0.08±0.01^b^	1.00±0.19^d^	4220 [3820–4665]

*Mean concentrations and standard deviations from the mean of five technical replicates are shown. Total detected flavonoid is the sum of the five listed flavonoids. Letters represent Tukey’s range test results after one way ANOVA for each metabolite or total detected flavonoids. Genotypes not sharing letters indicate statistically significant differences (*p* < 0.05). EC_50_ is the 50% effective concentration of extract needed to inhibit growth of the *Plasmodium falciparum* parasites. Artemisinin concentrations have been normalized to 5 mg/ml in wild type, *chi1-1* het, *chi1-1* hom and *cyp71av1-1* as detailed in section “Whole-Leaf Extraction for *P. falciparum* Kill Rate Assays.”*

### Artemisinin-Reduced Whole-Leaf Extracts Lack Potent and Rapid Antiplasmodial Activity

To test for the potential antiplasmodial activity of artemisinin-unrelated compounds in *A. annua*, we used the artemisinin-reduced *AMS* silenced plant line (Catania et al., 2018). Samples from this line were prepared alongside the other genetic variants and re-suspended to match the wild-type casticin levels, which resulted in a 100-fold reduction in artemisinin levels compared to the wild type (Table 1). Determination of the EC_50_ in the *AMS* silenced line revealed a greater than 20-fold reduction in potency when compared to the wild-type (Figure 3A). Moreover, samples from the *AMS* silenced line in the BRRoK assay lacked the rapid initial cytocidal activity of the wild type and heterozygous and homozygous *chi1-1* samples and were only apparently cytocidal at concentrations above 3xEC_50_ (Figure 3B).

We also used a *cyp71av1-1* mutant shown to be completely deficient in the synthesis of artemisinin (Czechowski et al., 2016) to investigate potential antiplasmodial effects of flavonoids (and other *A. annua* compounds) in the absence of artemisinin. As a control we used heterozygote *cyp71av1-1* that accumulates wild-type artemisinin levels. In extracts from *cyp71av1-1* antiplasmodial activity was reduced ∼300 fold compared to extracts from heterozygous *cyp71av1-1* (Figure 3C). The initial cytocidal activity of the control heterozygote *cyp71av1-1* extracts were comparable to those of the wild type and *chi1-1* extracts, whereas cytocidal activity was reduced in the *cyp71av1-1* mutant (Figure 3D). It is noteworthy that extracts from *cyp71av1*-1 homozygous lines are among the highest in total flavonoid content of the material used for anti-plasmodial assays (Table 1). Taken together these results represent convincing evidence that *A. annua* flavonoids do not exhibit anti-plasmodial activity in *in vitro* assays. These results also suggest that the sesquiterpene epoxide artennuin X, one of the most abundant metabolites produced by *cyp71av1-1* in the absence of artemisinin (Czechowski et al., 2016), also does not have appreciable antiplasmodial activity. This is not really surprising as arteannuin X does not carry an endoperoxide bridge (Czechowski et al., 2016), which is thought to be crucial for antiplasmodial activity of sesquiterpene lactones such as artemisinin.

## Discussion

### CHI1 Is Necessary for Trichome-Specific Flavonoid Synthesis

We report the identification and characterization of an *A. annua* mutant in *CHI1*, which encodes the enzyme that catalyzes the second committed step of the flavonoid biosynthesis pathway. The *chi1-1* mutation is predicted to result in a truncation that would preclude a sizable portion of the CHI1 functional monomer, including sections that may interact with the product naringenin (Figure 1C and Supplementary Figure S1A). Indeed, *chi1-1* failed to produce all four major polymethoxylated flavonoids, usually detected in young, mature and dry *A. annua* leaves (Figures 2Ai–iv). Flavonoid levels in heterozygous *chi1-1* were comparable with wild type (Artemis), which indicates that *chi1-1* is a recessive mutation (Figures 2Ai–iv). Expression profiling in various tissues of wild-type *A. annua* demonstrated that *CHI1* seems to be specifically expressed in trichomes (Figure 1A). In fact, we showed that the effect of the *chi1-1* mutation on metabolite levels is clearly trichome-specific (Figures 2B–D and Supplementary Table S2) which is consistent with the *CHI1* expression pattern (Figure 1A). The fact that two other *CHI* gene homologs expressed in *A. annua* (*CHI2* and *CHI3*) did not compensate for the lack of flavonoids in trichomes of *chi1-1* strongly suggests that CHI1 is the main enzyme responsible for flavonoid biosynthesis in *A. annua* trichomes.

The precursors of all secondary or specialized metabolites in higher plants are derived from primary metabolism. Phenylpropanoid biosynthesis leading to flavonoids relies on the synthesis of L-phenylalanine from chorismate, sourcing carbon precursors from the pentose phosphate pathway of primary metabolism. Terpenoid biosynthesis on the other hand starts from the common precursors supplied by the plastidic MEP and the cytosolic mevalonate pathways, which both rely on carbon sourced from glycolysis. Crosstalk between the phenylpropanoid and terpenoid biosynthetic pathways occurs, therefore, at the level of early carbon precursors, such as glyceraldehyde 3-phosphate and acetyl-CoA, and with reducing power provided by NAD(P)H and energy released from ATP hydrolysis. We had therefore speculated that artemisinin biosynthesis may be improved by specific blockage of flavonoid biosynthesis in *A. annua* trichomes, due to more carbon precursors becoming available for farnesyl pyrophosphate biosynthesis. However, we did not observe any increase in levels of artemisinin or related precursors in homozygous *chi1-1* mutants disrupted in flavonoid production (Figures 2Aiv,Bi). On the contrary, artemisinin levels in all *chi1-1* leaf ages were lower when compared to heterozygous *chi1-1* and the wild type (Figures 2Aiv,Bi). The reduction of artemisinin levels in *chi1-1* might be explained by lower DHAA levels (Supplementary Table S1), which could be due to either decreased DHAA synthesis or enhanced DHAA degradation, but the connection to the *chi1-1* mutation is unclear. The crosstalk between phenylpropanoid and terpenoid metabolism is further highlighted by the report that overexpression of the *A. annua CINNAMYL ALCOHOL DEHYDROGENASE* results in an increase in lignin and coumarin and a reduction in artemisinin and other sesquiterpenes (Ma et al., 2018).

### Flavonoids Had No Effect on the *in vitro* Antiplasmodial Activity of *A. annua* Extracts

Flavonoids have been suggested as candidates for increasing antiplasmodial activity and potentially slowing the emergence of resistance in whole-plant preparations, relative to artemisinin alone (Weathers et al., 2014; Elfawal et al., 2015). It has been proposed that these attributes may arise due to flavonoids enhancing artemisinin action by increasing artemisinin solubility in water (Mueller et al., 2000) or through the action of some flavonoids, such as casticin, in increasing artemisinin binding to hemin, one potential target of artemisinin action (Bilia et al., 2002). Artemisinin action *in vitro* against intraerythrocytic stages of *P. falciparum* typically provides an EC_50_ of 3–5 nM (Liu et al., 1992; Hasenkamp et al., 2013). Casticin, the most abundant flavonoid in *A. annua*, has an EC_50_ of 65 μM and 5 μM casticin reduced the artemisinin EC_50_ some 3–5 fold (Liu et al., 1992). Artemetin also reduces the artemisinin EC_50_, although to a lesser degree than casticin (Elford et al., 1987). In another report, the flavonoids artemetin, casticin, chrysoplenetin, chrysosplenol-D, cirsilineol and eupatorin have an IC_50_ that is 100 times that of artemisinin (Liu et al., 1992). When combining 5 μM of these flavonoids with artemisinin, the artemisinin IC_50_ is reduced to as much as half (Liu et al., 1992). However, the interactive mode of action of these compounds is unclear. In an isobologram analysis of compound interactions, casticin has an antagonistic antimalarial activity with artemisinin in a 3:1 combination (Suberu et al., 2013) but is apparently synergistic at a 10–10,000:1 combination (Elford et al., 1987; Liu et al., 1992). Therefore, additional compounds in whole-plant preparations could have synergistic or antagonistic effects with artemisinin depending on the relative concentration in the plant. Results of our *in vitro* antiplasmodial activity assays using *Artemisia* whole-leaf preparations do not show any synergistic effects between flavonoids and artemisinin, in contrast to previous reports (Ferreira et al., 2010; Suberu et al., 2013). We observed no appreciable differences between the artemisinin-producing heterozygous *chi1-1* (flavonoid containing) and homozygous *chi1-1* (flavonoid lacking) in terms of their EC_50_ potency or initial rate of cytocidal activity (Figure 3B). We therefore conclude that flavonoids do not appreciably contribute to the *in vitro* antiplasmodial activity beyond that provided by the artemisinin content, at least in the concentrations at which they are present in leaves of Artemis, a commercial F1 hybrid of *A. annua* (Delabays et al., 2001).

### The *in vitro* Antimalarial Activity of *A. annua* Extracts Is Predominantly Due to Artemisinin

Several groups have investigated compounds in *A. annua* extracts to find new sources of antimalarial activities other than artemisinin, or explore the possibility that *A. annua* compounds aid artemisinin (O’Neill et al., 1985; Elford et al., 1987; Liu et al., 1989, 1992; Mueller et al., 2000; Bhakuni et al., 2001). *A. annua* compounds having antimalarial activity have been reported but with EC_50_ values that are over three orders of magnitude higher than artemisinin (Suberu et al., 2013). In *in vitro* assays, arteannuin B and artemisinic acid have been shown to have additive antimalarial activity with artemisinin, whereas DHAA has antagonistic antimalarial activity with artemisinin (Suberu et al., 2013). Furthermore, some artemisinin precursors isolated from *A. annua* tea, including 9-epi-artemisinin and artemisitene, while being reported to have antimalarial activity themselves, can act antagonistically with artemisinin, possibly because they could have similar molecular targets in the malarial parasite (Suberu et al., 2013). However, artemisinin related compounds reported to either act by themselves or aid artemisinin are present in *A. annua* at much lower concentrations than required for antimalarial activity based on the EC_50_ (Elford et al., 1987; Bhakuni et al., 2001; Suberu et al., 2013), and therefore would perhaps not be expected to have an effect in whole-leaf extracts.

Our data suggests that the artemisinin-reduced extracts prepared so that they have wild-type casticin levels (Table 1), and likely the same concentration of non-artemisinin related compounds as wild-type extracts, had no *in vitro* antiplasmodial activity beyond that provided by the residual artemisinin in the homozygous *chi1-1* extracts (Figures 3A,B). We extended our studies to include the use of *cyp71av1-1* mutant extracts, which has been shown to completely lack artemisinin (Czechowski et al., 2016). Whereas the *cyp71av1-1* heterozygote control extract was essentially indistinguishable from those of the wild type and the *chi1-1* homozygote (Figures 3C,D), extracts of the *cyp71av1-1* homozygote were some 350–1000 fold less potent in their antiplasmodial activity. Whilst the *cyp71av1-1* homozygote did demonstrate a moderate to good initial cytocidal activity (Figure 3D), the BRRoK assay of these extracts used at least 10 times a greater concentration of extract than any other sample by virtue of these assays using multiples of the EC_50_.

While our results clearly demonstrate that flavonoids from *A. annua* plant extracts do not play a role in enhancing antiplasmodial activity relative to artemisinin in *in vitro* assays, the possibility remains that these compounds could have *in vivo* effects (Elfawal et al., 2012, 2015). It has been postulated that flavonoids could increase artemisinin solubility or inhibit activity of the cytochrome P450s responsible for degradation of artemisinin (Elfawal et al., 2012). *A. annua* extracts have been shown to result in higher artemisinin concentration in mice blood than the same concentration of artemisinin alone and this effect was attributed to arteannuin B (Cai et al., 2017). However, it should be noted that artemisinin is known to dissolve poorly in water and has a short serum half-life (Elfawal et al., 2012). Consequently, artemisinin is typically chemically converted to dihydroartemisinin, artesunate or artemether to improve solubility and increase its half-life in human serum (Petersen et al., 2011). These improved artemisinin-based compounds are combined with a companion drug from a different class to formulate ACTs - the WHO recommended method of treatment for patients with malaria. Companion drugs include lumefantrine, mefloquine, amodiaquine, sulfadoxine/pyrimethamine, piperaquine and chlorproguanil/dapsone. This combination contributes to high efficacy, fast action and reduction in the likelihood of resistance developing for ACTs. *In vivo* investigations into the effectiveness of whole plant extracts for the treatment of malaria should use approved artemisinin-related compounds with improved solubility and lifetime in human serum or indeed ACTs, rather than artemisinin alone, as a proper comparator in studies to investigate the potential of whole-leaf extracts from *A. annua*. We conclude that endogenous flavonoids present in whole-leaf extracts of *A. annua* have no appreciable effect on the antimalarial activity of artemisinin as determined by quantitative *in vitro* assays.

## Data Availability

All datasets generated for this study are included in the manuscript and/or the Supplementary Files.

## Author Contributions

TC, MR, DR, TW, TL, PH, and IG conceived and designed the research. TC, MR, DR, TW, DH, MF, and MV performed the experiments. TC, MR, TL, MF, MV, PH, and IG analyzed the data. TC, MR, PH, and IG wrote the manuscript.

## Conflict of Interest Statement

The authors declare that the research was conducted in the absence of any commercial or financial relationships that could be construed as a potential conflict of interest.
