# Supplementary figures and images for: Flavonoid Versus Artemisinin Anti-malarial Activity in Artemisia annua Whole-Leaf Extracts
Source: Front Plant Sci. 2019 Jul 30;10:984. doi: 10.3389/fpls.2019.00984 (PMC6683762; doi:10.3389/fpls.2019.00984)

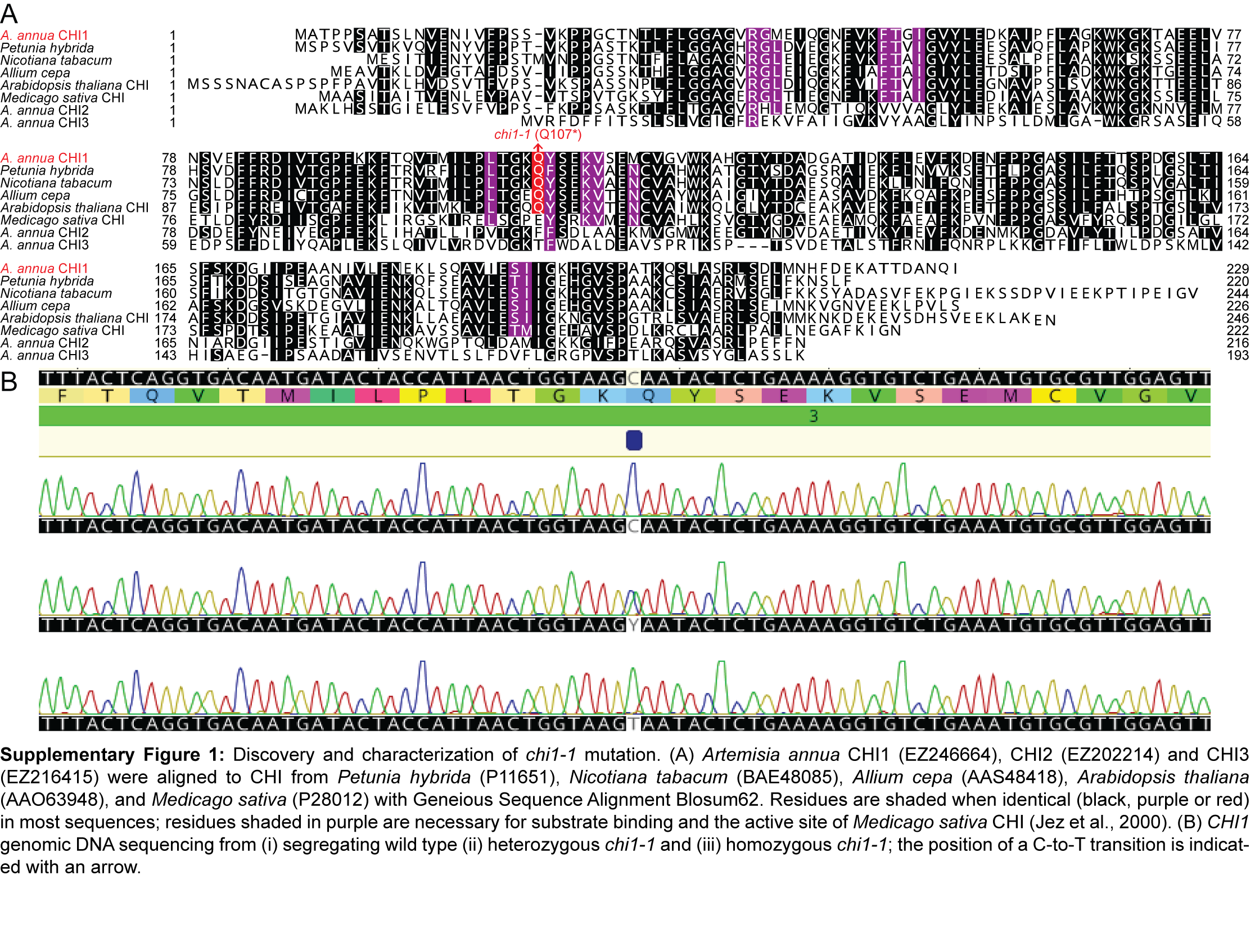

Supplement: Supplementary file 5 [file Image_1.tif]
